# Disease Module Identification Based on Representation Learning of Complex Networks Integrated From GWAS, eQTL Summaries, and Human Interactome

**DOI:** 10.3389/fbioe.2020.00418

**Published:** 2020-05-06

**Authors:** Tao Wang, Qidi Peng, Bo Liu, Yongzhuang Liu, Yadong Wang

**Affiliations:** School of Computer Science and Technology, Harbin Institute of Technology, Harbin, China

**Keywords:** disease module identification, GWAS, eQTL, node2vec, hierarchical clustering

## Abstract

The study of disease-relevant gene modules is one of the main methods to discover disease pathway and potential drug targets. Recent studies have found that most disease proteins tend to form many separate connected components and scatter across the protein-protein interaction network. However, most of the research on discovering disease modules are biased toward well-studied seed genes, which tend to extend seed genes into a single connected subnetwork. In this paper, we propose N2V-HC, an algorithm framework aiming to unbiasedly discover the scattered disease modules based on deep representation learning of integrated multi-layer biological networks. Our method first predicts disease associated genes based on summary data of Genome-wide Association Studies (GWAS) and expression Quantitative Trait Loci (eQTL) studies, and generates an integrated network on the basis of human interactome. The features of nodes in the network are then extracted by deep representation learning. Hierarchical clustering with dynamic tree cut methods are applied to discover the modules that are enriched with disease associated genes. The evaluation on real networks and simulated networks show that N2V-HC performs better than existing methods in network module discovery. Case studies on Parkinson's disease and Alzheimer's disease, show that N2V-HC can be used to discover biological meaningful modules related to the pathways underlying complex diseases.

## 1. Introduction

The genome-wide association studies (GWAS) have successfully identified vast of variants associated with complex diseases (Visscher et al., 2017). However, the gene targets responsible for GWAS signals largely remain elusive, which hinders the way of illuminating molecular mechanisms of complex diseases and developing novel drug targets (Gallagher and Chen-Plotkin, 2018; Cheng et al., 2019b). The challenge of transforming GWAS signals into clinical useful gene targets is mainly due to the fact that most susceptibility variants locate in non-coding regions and thus do not alter the protein sequence directly. Emerging evidence has shown that regulation of gene expression is important mechanism associated with disease susceptibility variants (Westra et al., 2013; GTEx Consortium, 2017; Watanabe et al., 2017). Thus, to understand the molecular mechanism underlying GWAS signals, there is an urgent need to investigate the genes regulated by disease-associated variants and gene modules which could be disturbed by these potential disease genes.

The development of genome-wide assay of genetic variants and gene expressions, makes it possible to systematically associate genetic variations with quantitive levels of gene expression in a population, which is known as expression quantitative trait loci (eQTL) analysis (GTEx Consortium, 2017). Advances in eQTL studies enable rapid identification of potential casual genes (i.e., eQTL regulated genes, egenes) genome-widely in relevant tissues of complex diseases (Fairfax et al., 2012; Cheng et al., 2018b; Dong et al., 2018; Wang et al., 2019a,b). The public available eQTL and other molecular signatures have become useful resources to nominate candidate casual genes of complex diseases (GTEx Consortium, 2017; Cheng et al., 2019a, 2020). However, the detailed understanding of the molecular mechanisms through which these egenes jointly affect disease phenotypes remains largely unclear, and their discovery is a challenging computational task (Cheng et al., 2019b; Peng et al., 2020a). Instead of analyzing binary relationships between single SNP and single gene, network-based analyses provide valuable insights into the higher-order structure of gene communities or pathways that those potential disease genes may work together in the etiology of complex diseases (Fagny et al., 2017; Cheng et al., 2019b; Peng et al., 2020b; Wang et al., 2020). And advances in deep learning and graph representation learning technologies improve the accuracy of identifying disease related biomarkers (Peng et al., 2019a,b). In this paper, our purpose is to derive disease related modules from an integrated network with multi-layer information including human interactome (mainly protein-protein interactions, PPI), and summaries of GWAS and eQTL studies. To aid this purpose, we present a novel algorithm named N2V-HC, which could learn deep representing features of nodes in the integrated molecular network, and unbiasedly detect gene communities enriched with potential disease genes (i.e., egenes in the context).

The identification of disease modules is driven by the primary observation that disease-related proteins tend to interact closely in biological network (Agrawal et al., 2018). In recent years, many studies have applied network-based methodologies to predict disease modules (Califano et al., 2012; Mäkinen et al., 2014; Ghiassian et al., 2015; Sharma et al., 2015; Calabrese et al., 2017). However, there are several challenges in current disease modules detection methods: (1) most methods rely on seed genes to expand the connected module. They adapt “seed-extend” strategy, starting from the well-studied disease genes and expanding the module by adding directly connected neighborhood. However, some complex diseases have no or only a few known disease genes, such as neurodegenerative disorders (e.g., Parkinson's disease, Alzheimer's disease etc.). This makes the process biased toward well-studied disease genes, and the discovery ability is largely limited by selected seed genes. (2) Recent studies have shown that most disease pathways do not correspond to single well-connected component in PPI network. Instead, disease proteins tend to form many separate connected components and scatter across the network (Agrawal et al., 2018). However, current methods tend to extend the seed genes into a large connected component or sub-network which might be less sufficient for discovering global disease modules. (3) The principle of node similarity measurement in current methods is mainly based on homophily, while ignoring the structural equivalence. Under the homophily hypothesis, nodes in the same module have higher similarity while under the structural equivalence hypothesis, nodes that have similar structural roles in network also have higher similarity. Studies have shown that the structural equivalence is also an important feature in measuring node similarity (Perozzi et al., 2014; Grover and Leskovec, 2016), which should also be considered.

To solve these challenges, our proposed method, N2V-HC, first predicts the disease genes based on genetic associations from summaries of GWAS and eQTL studies and integrates eQTL SNP (eSNP), eQTL regulated gene (egene) with human interactome network. Second, we use node2vec (Grover and Leskovec, 2016), an advanced network embedding method, to learn node features through a biased random walk process. The embedding process considers both the homophily and structural equivalence of nodes in the network. Third, nodes are clustered based on their embedding features using an iterative hierarchical clustering strategy. Modules are determined by a dynamic tree-cut strategy, and candidate disease modules are prioritized by evaluating whether the module is enriched for predicted disease genes. To evaluate the clustering performance of N2V-HC, we compared it with several state-of-the-art graph clustering methods including Markov clustering (MCL) (Enright et al., 2002), affinity propagation (AP) (Frey and Dueck, 2007), spectral clustering (Shi and Malik, 2000), mCODE (Bader and Hogue, 2003), GLay (Su et al., 2010), and hierarchical clustering on several real-world networks with ground truth labels, and also on multiple simulated networks. The experimental results showed that our method has better clustering performance than compared methods. We also performed case studies on Parkinson's disease (PD) and Alzheimer's disease (AD), and found biological meaningful modules associated with PD and AD, which might help to explain the pathology of diseases.

## 2. Methods

### 2.1. Overview

**Figure 1:**
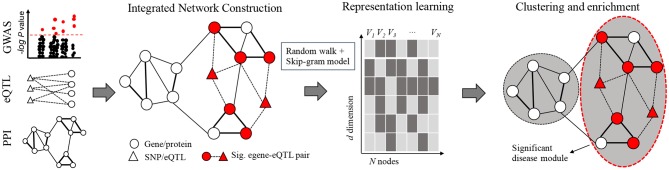
Framework of N2V-HC algorithm. The left-most panel shows input data sources of the integrated network: summary statistics of GWAS and eQTL studies, and PPI network or other types of networks. The edge width represents weight on edge. Representation learning step extracts global connectivity features for *N* nodes of the integrated network by using a biased random walk technology and the Skip-gram model. Each feature is a numeric vector of *d* dimension. Unsupervised hierarchical clustering method and dynamic tree-cut method are applied in an iterative module convergence process. The circle with red dash line represents the disease module which is significantly enriched with egenes.

In order to pinpoint key disease related modules, we propose a novel algorithm named N2V-HC, which could learn global connectivity features for nodes in an integrated molecular network, and automatically detect gene communities enriched with potential disease genes. The N2V-HC algorithm mainly consists of three steps as shown in Figure 1. Step 1: construction of integrated complex network. The integrated network is constructed based on known experimental molecular interaction networks, such as PPI network, and additional edges are added based on disease relevant signals from GWAS and the eQTL links between GWAS signals to network genes (section 2.2). Step 2: representation learning in network. N2V-HC learns features or embeddings for each node in the network by using node2vec (section 2.3). Step 3: identification of disease modules. Unsupervised hierarchical clustering method and dynamic tree-cut method are applied to partition network nodes into modules, and an iterative module convergence strategy is used. The disease module is finally prioritized by enrichment performance (section 2.4). Other methods are also detailed here (sections 2.5–2.7).

### 2.2. Construction of Integrated Complex Network

We project the eQTLs significantly associated with specific disease onto a gene interaction network, i.e., a PPI network in this work, and generate an integrated biological complex network, where disease modules are discovered. To make the network construction procedures more clear, we use susceptibility variants of Parkinson's disease (PD) and Alzheimer's disease (AD) as cases to illustrate the whole process.

#### 2.2.1. GWAS Data Preparation

First, we extract GWAS index SNPs of PD and AD from the most recent and largest GWAS papers conducted by Nalls et al. (2019) and Jansen et al. (2019). Second, we calculate proxy SNPs in linkage disequilibrium (LD) with index SNPs by setting LD *R*^2^≥0.6 using EUR population of 1000G genome reference panel (Genomes Project Consortium, 2015). Proxy SNPs are derived separately for PD and AD using SNiPA platform (https://snipa.helmholtz-muenchen.de/snipa3/?task=proxy_search), and other parameters are set in default.

#### 2.2.2. eQTL Data Preparation

As eQTL and gene expression are tissue-specific and PD and AD are also relevant to brain tissue, we first download eQTL summaries of brain frontal cortex from GTEx portal (https://gtexportal.org/). Then, we extract associations involving those GWAS-derived SNPs (index SNPs and their proxies). FDR is calculated based on the nominal *P*-values of the extracted eQTL associations. We use *FDR* ≤ 0.05 as cutoff to determine significant eQTL-egene associations.

#### 2.2.3. Human Interactome Preparation

First, we use the molecular physical interaction network complied by Menche et al. (2015), consisting of 141,296 physical interactions and 13,460 proteins. The edges of the network are experimentally documented in human cells, including protein-protein and regulatory interactions, metabolic pathway, and kinase-substrate interactions. Since some genes are not active in human brain, we filtered out 2,736 genes with low expression levels in frontal cortex based on the gene expression profiles in GTEx portal.

#### 2.2.4. Network Integration

We first projected the significant eQTL-egene pairs onto the human interactome. Since the input proxy SNPs can be tagged by index SNPs, we used the corresponding index SNPs to replace the proxy SNPs in the merged network.

### 2.3. Representation Learning of Network Structure

Node2vec (Grover and Leskovec, 2016) is applied to learn the global features or representations of nodes in the network. Node2vec is a network embedding method based on random walk, which has been successfully applied in bioinformatics applications (Grover and Leskovec, 2016; Cheng et al., 2018a). It learns node representations following two principles: nodes in the same community have similar embeddings (i.e., homophily); and nodes sharing similar structure roles have similar embeddings (i.e., structural equivalency).

Node2vec extends the Skip-gram model to networks. Given a graph *G* = (*V, E*), it learns the representation ${\vec{z}}_{u}=f\left(u\right)$ of node *u* by optimizing the objective function given by Equation 1, where *N*_*S*_(*u*) represents network neighborhood of node *u* generated by a sampling strategy *S*, and *f* : *V* → *R*^*n*×*d*^, where *d* is the dimension of the embedding space (i.e., the feature dimension of nodes). By making assumptions of conditional independence and symmetry of feature space, the objective function is further transformed into Equation (2).

(1) $$\begin{array}{r} \underset{f}{\max}\underset{u\in V}{\sum }\log P\left(N_{S}\left(u\right)|f\left(u\right)\right) \end{array}$$

(2) $$\begin{array}{r} \underset{f}{\max}\underset{u\in V}{\sum }\left\{-\log\left[\underset{v\in V}{\sum }\exp\left(f\left(u\right)\cdot f\left(v\right)\right)\right]+\underset{n_{i}\in N_{S}\left(u\right)}{\sum }f\left(n_{i}\right)\cdot f\left(u\right)\right\} \end{array}$$

In order to obtain the node neighborhood *N*_*S*_(*u*), node2vec uses a biased random walk method, which can perform flexible trade-offs between DFS and BFS. It calculates the node neighborhood by simulating a random walk of length *l*. Suppose the current position is node *v*, the previous position is node *t*, and the next step is to walk to node *x*. To determine the next node *x*, the transition probability is designed as shown in Equation (3), where α_*pq*_(*t, x*) is given by Equation (4) and *d*_*tx*_ = {0, 1, 2} represents the shortest path distance from node *t* to node *x*, and the *p* and *q* parameters constrain the direction of random walk (that is, a large *p* indicates closer to DFS, while a large *q* indicates closer to BFS). Let *c*_*i*_ represents the walker in step *i*, then the probability of visiting node *x* is given by Equation (5). Among them, *Z* represents a normalized constant, that is, $Z=\underset{\left(v,x\right)\in E}{\sum }\pi_{vx}$.

(3) $$\begin{array}{r} \pi_{vx}=\alpha_{pq}\left(t,x\right)\cdot w_{vx} \end{array}$$

(4) $$\begin{array}{r} \alpha_{pq}\left(t,x\right)=\{\begin{array}{c} \frac{1}{p},d_{tx}=0;\\ 1,d_{tx}=1;\\ \frac{1}{q},d_{tx}=2. \end{array} \end{array}$$

(5) $$\begin{array}{r} P\left(c_{i}=x|c_{i-1}=v\right)=\{\begin{array}{c} \frac{\pi_{vx}}{Z},\left(v,x\right)\in E;\\ 0,othersize. \end{array} \end{array}$$

### 2.4. Identification of Disease Modules

#### 2.4.1. Hierarchical Clustering and Dynamic Dendrogram-Cut

After learning the global connectivity features for each node in the network, we perform bottom-up hierarchical clustering to distinct modules. The hierarchical clustering initially treats each node as a cluster, and then iteratively merges the two clusters that have best similarity until the last one. Typically, N2V-HC uses Euclidean distance and average linkage method by default to construct the dendrogram. Then we apply Dynamic Hybrid tree-cut method on the dendrogram to obtain a flexible number of clusters.

The Dynamic Hybrid tree-cut method adopts bottom-up merging strategy (Langfeldera et al., 2008). Let *N* be the total number of nodes in a cluster, and *N*_0_ be the minimum number of nodes in a cluster. The cluster core is defined as the lowest *N*_*c*_ nodes in the cluster, where $N_{c}=\min\left\{int\left(\frac{N_{0}}{2}+\sqrt{N-\frac{N_{0}}{2}}\right),N\right\}$. The core scatter $\bar{d}$ is the average dissimilarity of the node pairs in the cluster core. The cluster gap *g* is the difference between $\bar{d}$ and the height of the cluster. The first step of the “Dynamic Hybrid” method is to merge the nodes/branches in the dendrogram bottom to up to get initial clusters. These clusters should satisfy the following four conditions: (1) *N* > *N*_0_; (2) the height of the cluster is less than the maximum tree height *h*_*max*_; (3) the cluster's core scatter $\bar{d}<d_{max}$; (4) The cluster gap *g* > *g*_*min*_. (*N*_0_, *h*_*max*_, *d*_*max*_, *g*_*min*_) can be specified by the user. This will leave out some single nodes or tiny clusters (cluster that meet the above conditions except *N* > *N*_0_), which are called outliers. The second step is to merge these outlier into the clusters generated in the first step. For these outliers, the outlier-cluster dissimilarity is calculated one by one, and is classified into the cluster most similar to it (Langfeldera et al., 2008).

#### 2.4.2. Iterative Module Selection Process

**Figure 2:**
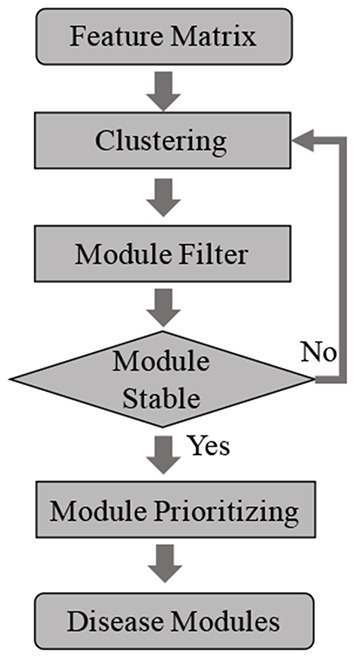
Steps of disease module identification.

After global clustering, the initial clusters are generated, some of which may be enriched with disease associated egenes, while other may not consist of any egenes. To boil down the number of candidate modules, we filter out modules that do not consist of any disease relevant egenes. The genes in left modules are then extracted as a subnetwork, and we repeat the clustering and dynamic dendrogram-cut processes. These steps will be iteratively performed until the modules are stable, which means current clustering results stay same with last clustering results. After the process is convergent, all left modules consist of disease relevant egenes, which are the candidate disease modules. The iterative module selection process is shown in Figure 2.

#### 2.4.3. Prioritizing Disease Modules by Enrichment Analysis

We then test whether egenes are enriched in the candidate disease modules. The enrichment analysis is performed by Fisher's exact test. All genes shown in the network with size *n* are used as background genes, and are assigned to four cells of a two by two contingency table, according to if a gene is in a module or not, and if it is a egene or not. For example, given a module *M*, suppose *a* is the number of genes that are in module *M* and are egenes; *b* represents the number of genes that are egenes but not in *M*; *c* is number of genes in module *M*, but are not egenes; *d* represents number of genes that are not egenes and not in module *M*, the fisher's exact test *P*-value is given by the Equation 6:

(6) P=(a+ba)(c+dc)(na+c)

### 2.5. Module Mapping

To evaluate the performance of module detection on ground truth datasets or simulated datasets, it is essential to first match the modules discovered by methods under evaluation with the ground truth modules. We model this module mapping problem by a classical task assignment algorithm. The task assignment problem is a fundamental combinatorial optimization problem. Suppose there are *N* agents and *N* tasks, each agent will be assigned to perform a task, and there will be a cost generated for each agent-task assignment, the object is to find the best task assignment strategy to minimize the cost. In context of the module mapping problem, our purpose is to find the best bijection between predicted module set and ground truth module set, which maximize the size of module intersections. In formula, let the intersection matrix as {*S*_*i, j*_}_N*N_, where *s*_*i, j*_ = 1 represents the number of overlapping nodes between module *i* and module *j*, and the binary matrix as {*M*_*i, j*_}_N*N_, where *m*_*i, j*_ = 1 if and only if module *i* is matched with module *j*, otherwise *m*_*i, j*_ = 0. To guarantee one-to-one correspondence, two conditions are needed: $\overset{N}{\underset{i=1}{\sum }}m_{i,j}=1$ and $\overset{N}{\underset{j=1}{\sum }}m_{i,j}=1$. The objective is to optimize the binary matching matrix {_*M*_*i, j*_}N*N_ which maximizes $\overset{N}{\underset{i=1}{\sum }}\overset{N}{\underset{j=1}{\sum }}s_{i,j}*m_{i,j}$.

In addition, there is a common case that the number of predicted modules is not equal to the module number in ground truth. And this is an unbalanced task assignment problem. As a solution, we manually add empty modules to the short module sequence, to make sure the two module sequences have same length. Then, the problem is transformed to balanced task assignment problem, as described above.

### 2.6. Micro F1 Score

In binary classification problem, the F1 score is commonly used performance indicator, as shown in Equation (7), where $precision=\frac{TP}{TP+FP}$, and $recall=\frac{TP}{TP+FN}$.

(7) $$\begin{array}{r} F1=\frac{2*precision*recall}{precision+recall} \end{array}$$

The module detection is similar to multi-label classification problem. To compare the module detection performance of different methods on ground truth datasets, we use micro F1 score as the indicator. The micro F1 score is a variant of F1 score, as shown in Equation (8), where *precision*_*Micro*_ is defined in Equation (9) and *recall*_*Micro*_ is defined in Equation (10). Suppose there are *N* predicted modules, the *TP*_*i*_, *FP*_*i*_, *FN*_*i*_ in Equations (9) and (10) represent the number of true positive nodes, false positive nodes and false negative nodes in module *i*, respectively.

(8) $$\begin{array}{r} F1_{Micro}=\frac{2*recall_{Micro}*precision_{Micro}}{recall_{Micro}+precision_{Micro}} \end{array}$$

(9) $$\begin{array}{r} precision_{Micro}=\frac{\sum_{i=1}^{N}TP_{i}}{\sum_{i=1}^{N}\left(TP_{i}+FP_{i}\right)} \end{array}$$

(10) $$\begin{array}{r} recall_{Micro}=\frac{\sum_{i=1}^{N}TP_{i}}{\sum_{i=1}^{N}\left(TP_{i}+FN_{i}\right)} \end{array}$$

### 2.7. Gene Set Enrichment Analysis

Gene enrichment analysis is performed by overlapping genes in a module with Gene Ontology (GO) gene sets using GSEA with the C2 and C5 collection of the MSigDB. Genes shown in candidate disease modules are mapped onto MSigDB and are evaluated by fisher's exact test. The top 50 significantly enriched terms are used.

## 3. Results and Discussion

The accuracy of disease module detection in N2V-HC largely depends on the unsupervised clustering process. In this section, we first compared N2V-HC with several classical graph clustering methods, including Affinity propagation, GLay, MCL, Spectral clustering, mCODE, and Hierarchical clustering on various types of testing networks with labels of ground truth modules. Next, we applied N2V-HC to Parkinson's disease and Alzheimer's disease with PPI network, the latest GWAS summaries and brain eQTL summaries. We found (1) our method significantly performs better than compared methods; (2) most of the identified disease modules correspond to core disease-relevant pathways, which often comprise therapeutic targets.

### 3.1. Clustering Performance on Real-World Networks

To evaluate the clustering performance of N2V-HC, we compared it with several state-of-the-art graph clustering methods, including Markov clustering (MCL) (Enright et al., 2002), affinity propagation (AP) (Frey and Dueck, 2007), spectral clustering (Shi and Malik, 2000), mCODE (Bader and Hogue, 2003), GLay (Su et al., 2010), and hierarchical clustering. Six real-world networks with various sizes, densities, types (weighted/unweighted, directed/undirected) and ground truth cluster labels were used as testing datasets, including: Zachary's karate club network (Zachary, 1977), UKfaculty social network (Nepusz et al., 2008), Dolphin Social Network (Lusseau et al., 2003), College football game network (Girvan and Newman, 2002), US Political Books network (Krebs, 2004), and Cora citation network (Fakhraei et al., 2015). The six real-world networks are summarized in Table 1.

**Table 1:** Summary of real-world network datasets.

**Dataset**	**#Nodes**	**#Edges**	**Density**	**#Clusters**	**Graph type**	**References**
Karate	34	78	1.4E-1	2	w, ud	Zachary, 1977
Dolphins	62	159	8.4E-2	2	uw, ud	Lusseau et al., 2003
UKfaculty	81	817	2.5E-1	4	w, ud	Nepusz et al., 2008
Polbooks	105	441	8.1E-2	3	uw, ud	Krebs, 2004
Football	115	613	9.4E-2	12	uw, ud	Girvan and Newman, 2002
Cora	2,708	5,429	1.4E-3	7	uw, ud	Fakhraei et al., 2015

*w, weighted graph; uw, unweighted graph; ud, undirected graph*.

To evaluate their performance, micro F1 score was chosen as the indicator of performance (see section 2). We first map the predicted modules with ground truth modules by maximizing the overlap size of all modules (see section 2). Then, true positive (TP), false positive (FP), true negative (TN) and false negative (FN) number of nodes in each predicted module were calculated and leveraged into the micro F1 score (see section 2). To be noted, we fine-tuned the corresponding parameters of N2V-HC and compared methods to make the number of predicted modules close to the true module numbers. The experiment results were summarized in Table 2. As we can see, our method performs significantly better than most compared methods in the six real-world networks.

**Table 2:** Clustering performance on real-world networks.

**Datasets**	**AP**	**GLay**	**MCL**	**SC**	**HC**	**mCODE**	**N2V-HC ****(*MMS, DS, NPC*)******
Karate	0.844	0.847	0.529	0.588	0.588	0.623	**0.941** (10, 2, 2)
Dolphins	0.935	0.804	0.677	0.613	0.565	0.533	**0.984** (10, 2, 2)
UKfaculty	0.494	0.889	0.951	0.370	0.333	0.397	**0.963** (10, 2, 3)
Polbooks	0.609	0.816	0.838	0.400	0.438	0.451	**0.848** (10, 2, 4)
Football	0.113	0.583	**0.930**	0.235	0.235	0.435	0.922 (5, 2, 11)
Cora	0.356	0.512	0.294	0.298	0.287	0.295	**0.661** (100, 0, 6)

*AP, affinity propagation; MCL, Markov cluster; SC, spectral clustering; HC, hierarchical clustering; MMS, minModuleSize; DS, DeepSplit; NPC, number of predicted clusters. Parameter setting: MCL inflation factor setting: Karate 2.0, Dolphins 2.0, UKfaculty 2.5, Polbooks 2.1, Football 2.0, Cora 1.8. Parameters in AP, GLay, SC, HC, and mCODE were in default except that cluster number was set to the ground truth if available. Bold Values indicate the best micro F1 scores*.

**Figure 3:**
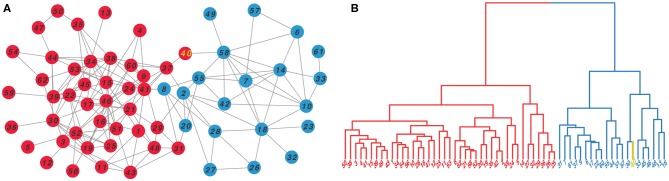
The clustering effect of N2V-HC on Dolphins social network (Lusseau et al., 2003). **(A)** The topology of original network, with colors represents the ground truth communities. **(B)** The hierarchical clustering dendrogram constructed by N2V-HC, where each leaf node represents a member in original network. Two predicted modules are colored in red and blue. Node “40,” which is misclassified, is labeled in yellow.

As a case, we illustrated the clustering effect of N2V-HC on Dolphins social network as shown in Figure 3. The original Dolphins social network is shown on the left panel, with red and blue colors representing two ground truth modules. The right panel shows the hierarchical dendrogram constructed by N2V-HC, where the leaf nodes represent the original dolphin members in the network, and the two predicted modules are also colored in red and blue. Only one node, with label “40,” is wrongly classified into opposite module, which is colored in yellow. However, we can see from the original network, the node “40” actually appears at the border of both modules, and could be arbitrarily classified.

### 3.2. Clustering Performance on Simulated Networks

We then evaluated the performance of N2V-HC on simulated networks in various scales. We used the network simulation tool LFR-benchmark (Lancichinetti et al., 2008), to generate small-to-large scale networks, with weighted and directed edges. The character of simulated networks can be adjusted by function LFR(*N*, *k*, *maxk*, *muw*, *t*1, *t*2), where *N* controls the number of network nodes, *k* controls the average degree of the node, *maxk* controls the maximum degree of the node, *muw* controls the mixing parameter for the weight, *t*1 controls minus exponent for the degree sequence, and *t*2 controls minus exponent for the community size distribution. We set *muw* = 0.5, *t*1 = 2, *t*2 = 1 in their default values. By setting different combination of parameters *N*, *k*, and *maxk*, we generated five networks in different scales (shown in Table 3). Then we run N2V-HC and compared methods on these five networks, the resulting micro F1 score is shown in Table 4. We can see that N2V-HC still performs much better than compared methods in different schema. With the network getting larger and more complex, the performance of compared methods tend to dramatically decline, while our method has better stability, indicating the robustness of N2V-HC. Combining the above experiments, we can conclude that N2V-HC can accurately extract the intrinsic network modules, which enables the ability to predict disease-relevant modules.

**Table 3:** Summary of LFR simulated networks.

**LFR(N, k, maxk)**	**Nodes**	**Edges**	**Density**	**Clusters**
LFR (100, 10, 30)	100	1,047	0.212	7
LFR (500, 10, 50)	500	5,269	0.042	36
LFR (1000, 20, 100)	1,000	19,115	0.038	39
LFR (2000, 30, 200)	2,000	60,946	0.030	34

**Table 4:** Clustering performance on LFR-benchmark datasets.

**Datasets**	**AP**	**GLay**	**MCL**	**SC**	**HC**	**mCODE**	**N2V-HC****(*MMS, DS, NPC*)******
LFR (100, 10, 30)	0.304	0.131	0.350	0.28	0.26	0.35	**0.615** (6, 2, 8)
LFR (500, 10, 50)	0.090	0.127	0.120	0.128	0.14	0.138	**0.496** (4, 3, 38)
LFR (1,000, 20, 100)	0.097	0.075	**0.692**	0.103	0.109	0.145	0.620 (6, 3, 40)
LFR (2,000, 30, 200)	0.092	0.033	0.651	0.080	0.082	0.135	**0.682** (5, 2, 34)

*AP, affinity propagation; MCL, Markov cluster; SC, spectral clustering; HC, hierarchical clustering; MMS, minModuleSize; DS, DeepSplit; NPC, number of predicted clusters. Parameter setting: MCL inflation factor was set in default (2.5) for all networks. Parameters in AP, GLay, SC, HC, and mCODE were in default except that cluster number was set to the ground truth if available. Bold Values indicate the best micro F1 scores*.

### 3.3. Case Studies on Parkinson's Disease and Alzheimer's Disease

Alzheimer's disease and Parkinson's disease are the top two neurodegenerative disorders, whose etiological mechanisms are still unclear. To predict the disease-relevant modules, we first constructed the networks integrated from GWAS, eQTL data, and human interactome by following steps (see section 2): (1) 90 and 32 independent GWAS index SNPs were obtained from the latest largest-scale to date GWAS of PD (Nalls et al., 2019) and AD (Jansen et al., 2019), respectively. (2) 7,194 and 1,270 proxy SNPs were derived separately based on 1000G EUR population for PD and AD. (3) eQTL associations were extracted for those GWAS-derived SNPs (index SNPs and their proxies) from summaries of GTEx brain frontal cortex (version V7). After filtering by threshold *FDR* ≤ 0.05, 41,538 significant associations, representing 248 egenes and 4,821 eSNPs were extracted for PD; and 370 significant associations, representing 19 egenes and 150 eSNPs were extracted for AD. (4) We downloaded the molecular physical interaction network complied by Menche et al. (2015), which consists of 110,913 physical interactions and 10,724 proteins after removing genes with low expression levels in frontal cortex. (5) Finally, we projected the significant eQTL-egene pairs onto the human interactome. Since the input proxy SNPs can be tagged by index SNPs, we used the corresponding index SNPs to replace the proxy SNPs in the merged network. The outcome integrated network for PD consists of 10,912 nodes, including 10,852 genes and 60 independent PD susceptibility SNPs, and 111,038 edges. The outcome integrated network for AD consists of 10,736 nodes, including 10,727 genes and 9 independent AD susceptibility SNPs, and 110,803 edges. Then we performed N2V-HC on these two integrated networks, by setting the dimension of representing features as 128, and the Dynamic Hybrid tree-cut parameter as *minModuleSize* = 20 and *deepSplit* = 2.

For integrated network of PD, the module detection process converged after four iterations, resulting in 51 candidate disease modules containing at least one egene (Table S1). Fisher's exact test was conducted for each module to test whether egenes were over-expressed in the module. And FDR was calculated to evaluate the enrichment significance. After filtering by *FDR* ≤ 0.05, 15 modules were predicted as the PD disease modules, which on average covered 80 genes. We next investigated the module function by performing gene set enrichment analysis (GSEA) (Mootha et al., 2003; Subramanian et al., 2005). Specifically, we computed the overlaps between module genes and gene sets in C2 (curated gene sets) and C5 (GO gene sets) categories of MSigDB (Liberzon et al., 2015). Among the 15 predicted PD modules, 12 (80%) modules have been annotated with functions relevant to known PD pathways (Table 5, Table S1). For example, the cellular pathways including oxidative stress, immune response, endosomal-lysosomal dysfunction, intra-cellular trafficking stress etc., have been widely reported associated with PD pathology in literatures (Parker et al., 2008; Mosley et al., 2012; Dehay et al., 2013; Abeliovich and Gitler, 2016).

**Table 5:** Gene set enrichment analysis of PD modules.

**ID**	**# Gene**	**# PD egene**	***P*-value**	**FDR**	**GSEA inferred module function**	**PD-relevant evidence**
PD36	39	20	2.94E-23	1.50E-21	GPCR ligand binding	Martin et al., 2005
PD41	33	17	5.62E-20	9.55E-19	Retinoic acid biosynthesis	Jacobs et al., 2007; Esteves et al., 2015,
PD42	32	13	7.47E-14	9.52E-13	GPI-anchor biosynthesis, ER/Golgi trafficking, Membrane lipid biosynthesis	Wang et al., 2014, Abeliovich and Gitler, 2016
PD12	126	19	5.45E-11	5.56E-10	Endocytosis, Immune response	Mosley et al., 2012; Abeliovich and Gitler, 2016
PD20	80	13	2.57E-08	2.18E-07	Immune response, Integrin cell surface	Wu and Reddy, 2012
PD37	38	9	1.28E-07	9.35E-07	Potassium channels, Glycogen metabolism	Chen et al., 2018
PD44	30	7	3.75E-06	2.12E-05	Hemoglobin complex	Freed and Chakrabarti, 2016
PD10	135	13	1.18E-05	6.00E-05	Oxidoreductase activity	Parker et al., 2008
PD34	42	7	3.94E-05	1.82E-04	Glycosaminoglycans biosynthesis	Lehri-Boufala et al., 2015
PD45	29	5	4.43E-04	1.74E-03	Immune response, Natural killer cell mediated immunity	Mihara et al., 2008
PD35	42	5	2.49E-03	9.08E-03	Lysosome, Sphingolipic metabolism	Dehay et al., 2013, Lin et al., 2019
PD46	29	4	3.96E-03	1.34E-02	WNT signaling pathway, Dopaminergic neuron differentiation	Arenas, 2014

*# Gene, number of genes in a module; # PD egene, number of egene regulated by PD susceptibility variants in a module*.

Similarly, we also obtained eight candidate modules associated with AD, among which four modules had *FDR* ≤ 0.05 based on Fisher's exact test (Table 6, Table S2). These molecular pathways include immune response, WNT signaling pathway, JAK/SAT signaling pathway and intra-cellular trafficking, which also have been reported associated with AD pathology in literatures (dos Santos and Smidt, 2011; Nicolas et al., 2013; Placido et al., 2014; Wang et al., 2018). Interestingly, the predicted AD modules and PD modules have similar functions, for example, AD1, AD3, PD12, PD20, and PD45 are all associated with immune response; AD2 and PD46 are associated with WNT signaling pathway and dopaminergic neuron differentiation; AD4 and PD42 are associated with intracellular trafficking. Three module pairs have high similarity including (AD1, PD20), (AD2, PD46), and (AD4, PD34), whose intersection size and Jaccard index are (67, 0.68), (21, 0.44), and (18, 0.26), respectively. There is no similarity (Jaccard index = 0) or very low similarity (Jaccard index < 0.05) between other AD-PD module pairs. These evidence indicate that AD and PD might share remarkably similar dysregulated pathways; and multiple modules may work together in the same disease pathway (e.g., immune response), where shared modules might be involved between AD and PD pathology.

**Table 6:** Gene set enrichment analysis of AD modules.

**ID**	**# Gene**	**# AD egene**	***P*-value**	**FDR**	**GSEA inferred module function**	**AD-relevant evidence**
AD1	88	6	6.36E-09	5.09E-08	Immune response	Wang et al., 2018
AD2	42	3	5.16E-05	2.07E-04	WNT signaling pathway, Dopaminergic neuron differentiation	dos Santos and Smidt, 2011
AD3	177	4	2.28E-04	6.08E-04	Immune response, JAK/STAT signaling pathway	Nicolas et al., 2013
AD4	52	2	3.73E-03	7.47E-03	ER/Golgi trafficking, Glycosaminoglycans metabolism	Placido et al., 2014

**Figure 4:**
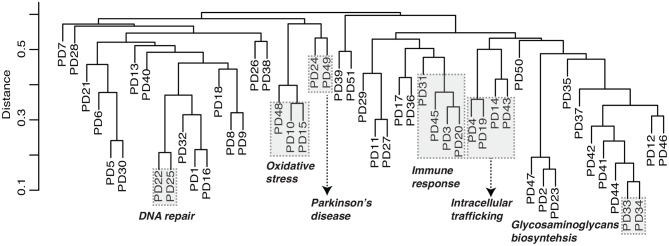
Module dendrogram of Parkinson's disease. Dendrogram of modules is built based on module eigen feature, i.e., the eigen vector corresponding with the first principle component of node features in a module. Distance is measured as one minus Pearson's correlation coefficient. Modules covered by the shaded rectangle share similar functions as illustrated.

In order to investigate the relationship between the predicted disease modules, our method is able to built the dendrogram of all candidate modules based on the module eigen feature, defined as the eigen vector of node features in a module corresponding with the first principle component. For example, the module dendrogram of Parkinson's disease was shown in Figure 4. We found several module blocks (modules with high similarity covered by shaded rectangle as shown in Figure 4) are annotated with similar functions. For example, PD10, PD15, and PD48 are related to oxidative stress; PD3, PD20, PD31, and PD45 are related to immune response; PD4, PD14, PD19, and PD43 are related to intracellular trafficking; PD33 and PD34 are related to glycosaminoglycans biosynthesis. Especially, PD24 and PD49 are both annotated as Parkinson's disease pathway (GSEA FDR = 1.2 * 10^128^ and 7 * 10^15^) and mitochondrial process (GSEA FDR = 5.7 * 10^141^ and 1.5 * 10^20^) by GSEA. The module dendrogram provide guidance to merge multiple modules into a super module, and can also be used to infer module functions.

As a secondary finding, we found some of the provisionally insignificant candidate modules were also associated with functions relevant to AD and PD pathology. For example, two modules were directly annotated as Parkinson's disease pathway (PD24, GSEA FDR = 1.2 * 10^128^) and Alzheimer's disease pathway (AD6, GSEA FDR = 2 * 10^8^). We also found modules associated with autophagy (PD13), apoptosis (PD1), post-synapse (PD11), SNARE binding (PD19), and mitochondria (PD15, PD48, PD49, PD9), which are believed to have played a role in PD etiology (Dehay et al., 2013; Abeliovich and Gitler, 2016).

Furthermore, our method generates disease modules without bias toward the seed genes. The traditional methods adapt “seed-extend” strategy, starting from the disease seed genes and expanding the module by adding neighborhood. For example, the DIAMOnD algorithm (Ghiassian et al., 2015) first defines the disease module as the subnetwork only consisting of the well-studied disease genes (seed genes). Next, for each iteration, one gene (named DIAMOnD gene) with highest connectivity score with the module will be added to grow the module, until all genes in the network are added. The first added *N* DIAMOnD genes (*N* is arbitrarily defined by user) together with the seed genes will form the final disease module. Thus, the module generated under “seed-extend” strategy is biased toward seed genes. However, in our N2V-HC method, the seed genes are masked during the hierarchical clustering procedure. In other words, our module generation process is not based on seed genes. Instead, we use seed genes as posterior knowledge to prioritize modules based on enrichment significance.

## 4. Conclusions

Disease module identification is often a crucial step to discover disease pathway and potential drug targets. In this article, we present a new algorithm framework, named N2V-HC, to predict disease modules based on deep feature learning of biological complex networks. Our method includes three steps: First, integrating a network from GWAS, eQTL summaries, and human interactome; Second, learning the node representing features in the integrated network; Third, detecting modules based on hierarchical clustering, and evaluating whether some of modules may be candidates for specific disease by determining their enrichment with egenes that are regulated by disease susceptibility variants. Experiments on network datasets with ground true labels suggest our method has better performance in module detection than compared methods. In addition, we apply N2V-HC on Parkinson's disease and Alzheimer's disease, and find significant modules associated with PD and AD. In general, our method can be used to incorporate with other types of networks beside PPI. We believe it will be a powerful tool for researchers to understand the molecular mechanisms of complex diseases in the post-GWAS era.

## Data Availability Statement

GTEx eQTL datasets can be downloaded at the GTEx portal (https://gtexportal.org/). The implementation of N2V-HC can be freely downloaded at Github (https://github.com/QidiPeng/N2V-HC).

## Author Contributions

TW designed the study, analyzed the data, and wrote the paper. QP implemented the algorithm framework, co-analyzed the data, and co-wrote the paper. BL, YL, and YW supervised the research, provided funding support, and revised the paper.

## Conflict of Interest

The authors declare that the research was conducted in the absence of any commercial or financial relationships that could be construed as a potential conflict of interest.

## References

[B1] Abeliovich A. Gitler A. D. (2016). Defects in trafficking bridge Parkinson's disease pathology and genetics. Nature 539, 207–216. 10.1038/nature20414 27830778

[B2] Agrawal M. Zitnik M. Leskovec J. (2018). “Large-scale analysis of disease pathways in the human interactome,” in PSB (Hawaii: World Scientific), 111–122. 10.1142/9789813235533_0011 PMC5731453 29218874

[B3] Arenas E. (2014). Wnt signaling in midbrain dopaminergic neuron development and regenerative medicine for Parkinson's disease. J. Mol. Cell Biol. 6, 42–53. 10.1093/jmcb/mju001 24431302

[B4] Bader G. D. Hogue C. W. (2003). An automated method for finding molecular complexes in large protein interaction networks. BMC Bioinformatics 4:2. 10.1186/1471-2105-4-2 12525261 PMC149346

[B5] Calabrese G. M. Mesner L. D. Stains J. P. Tommasini S. M. Horowitz M. C. Rosen C. J.. (2017). Integrating GWAS and co-expression network data identifies bone mineral density genes SPTBN1 and MARK3 and an osteoblast functional module. Cell Syst. 4, 46–59. 10.1016/j.cels.2016.10.014 27866947 PMC5269473

[B6] Califano A. Butte A. J. Friend S. Ideker T. Schadt E. (2012). Leveraging models of cell regulation and GWAS data in integrative network-based association studies. Nat. Genet. 44, 841–847. 10.1038/ng.2355 22836096 PMC3593099

[B7] Chen X. Xue B. Wang J. Liu H. Shi L. Xie J. (2018). Potassium channels: a potential therapeutic target for Parkinson's disease. Neurosci. Bull. 34, 341–348. 10.1007/s12264-017-0177-3 28884460 PMC5856711

[B8] Cheng L. Jiang Y. Ju H. Sun J. Peng J. Zhou M. Hu Y. (2018a). Infacront: calculating cross-ontology term similarities using information flow by a random walk. BMC Genomics 19:919. 10.1186/s12864-017-4338-6 29363423 PMC5780854

[B9] Cheng L. Qi C. Zhuang H. Fu T. Zhang X. (2020). gutMDisorder: a comprehensive database for dysbiosis of the gut microbiota in disorders and interventions. Nucleic Acids Res. 48, D554-D560. 10.1093/nar/gkz843 31584099 PMC6943049

[B10] Cheng L. Yang H. Zhao H. Pei X. Shi H. Sun J.. (2019a). MetSigDis: a manually curated resource for the metabolic signatures of diseases. Brief. Bioinformatics 20, 203–209. 10.1093/bib/bbx103 28968812

[B11] Cheng L. Zhao H. Wang P. Zhou W. Luo M. Li T.. (2019b). Computational methods for identifying similar diseases. Mol. Ther. Nucleic Acids. 18, 590–604. 10.1016/j.omtn.2019.09.019 31678735 PMC6838934

[B12] Cheng L. Zhuang H. Yang S. Jiang H. Wang S. Zhang J. (2018b). Exposing the causal effect of c-reactive protein on the risk of type 2 diabetes mellitus: a Mendelian randomization study. Front. Genet. 9:657. 10.3389/fgene.2018.00657 30619477 PMC6306438

[B13] Dehay B. Martinez-Vicente M. Caldwell G. A. Caldwell K. A. Yue Z. Cookson M. R.. (2013). Lysosomal impairment in Parkinson's disease. Mov. Disord. 28, 725–732. 10.1002/mds.25462 23580333 PMC5131721

[B14] Dong X. Liao Z. Gritsch D. Hadzhiev Y. Bai Y. Locascio J. J.. (2018). Enhancers active in dopamine neurons are a primary link between genetic variation and neuropsychiatric disease. Nat. Neurosci. 21, 1482–1492. 10.1038/s41593-018-0223-0 30224808 PMC6334654

[B15] dos Santos M. T. A. Smidt M. P. (2011). En1 and Wnt signaling in midbrain dopaminergic neuronal development. Neural Dev. 6:23 10.1186/1749-8104-6-23 21569278 PMC3104484

[B16] Enright A. J. Van Dongen S. Ouzounis C. A. (2002). An efficient algorithm for large-scale detection of protein families. Nucleic Acids Res. 30, 1575–1584. 10.1093/nar/30.7.1575 11917018 PMC101833

[B17] Esteves M. Cristóv ao A. C. Saraiva T. Rocha S. M. Baltazar G. Ferreira L.. (2015). Retinoic acid-loaded polymeric nanoparticles induce neuroprotection in a mouse model for Parkinson's disease. Front. Aging Neurosci. 7:20. 10.3389/fnagi.2015.00020 25798108 PMC4351630

[B18] Fagny M. Paulson J. N. Kuijjer M. L. Sonawane A. R. Chen C.-Y. Lopes-Ramos C. M.. (2017). Exploring regulation in tissues with eqtl networks. Proc. Natl. Acad. Sci. U.S.A. 114, E7841-E7850. 10.1073/pnas.1707375114 28851834 PMC5604022

[B19] Fairfax B. P. Makino S. Radhakrishnan J. Plant K. Leslie S. Dilthey A.. (2012). Genetics of gene expression in primary immune cells identifies cell type-specific master regulators and roles of HLA alleles. Nat. Genet. 44, 502–510. 10.1038/ng.2205 22446964 PMC3437404

[B20] Fakhraei S. Foulds J. Shashanka M. Getoor L. (2015). “Collective spammer detection in evolving multi-relational social networks,” in Proceedings of the 21th ACM SIGKDD International Conference on Knowledge Discovery and Data Mining (Sydney, NSW: ACM), 1769–1778. 10.1145/2783258.2788606

[B21] Freed J. Chakrabarti L. (2016). Defining a role for hemoglobin in Parkinson's disease. NPJ Parkinson's Dis. 2, 1–4. 10.1038/npjparkd.2016.21 28725702 PMC5516577

[B22] Frey B. J. Dueck D. (2007). Clustering by passing messages between data points. Science 315, 972–976. 10.1126/science.1136800 17218491

[B23] Gallagher M. D. Chen-Plotkin A. S. (2018). The post-GWAS era: from association to function. Am. J. Hum. Genet. 102, 717–730. 10.1016/j.ajhg.2018.04.002 29727686 PMC5986732

[B24] Genomes Project Consortium (2015). A global reference for human genetic variation. Nature 526, 68–74. 10.1038/nature15393 26432245 PMC4750478

[B25] Ghiassian S. D. Menche J. Barabási A.-L. (2015). A disease module detection (diamond) algorithm derived from a systematic analysis of connectivity patterns of disease proteins in the human interactome. PLoS Comput. Biol. 11:e1004120. 10.1371/journal.pcbi.1004120 25853560 PMC4390154

[B26] Girvan M. Newman M. E. (2002). Community structure in social and biological networks. Proc. Natl. Acad. Sci. U.S.A. 99, 7821–7826. 10.1073/pnas.122653799 12060727 PMC122977

[B27] Grover A. Leskovec J. (2016). “node2vec: Scalable feature learning for networks,” in Proceedings of the 22nd ACM SIGKDD International Conference on Knowledge Discovery and Data Mining (San Francisco, CA: ACM), 855–864. 10.1145/2939672.2939754 PMC5108654 27853626

[B28] GTEx Consortium (2017). Genetic effects on gene expression across human tissues. Nature 550, 204–213. 10.1038/nature24277 29022597 PMC5776756

[B29] Jacobs F. M. Smits S. M. Noorlander C. W. von Oerthel L. van der Linden A. J. Burbach J. P. H.. (2007). Retinoic acid counteracts developmental defects in the *Substantia nigra* caused by Pitx3 deficiency. Development 134, 2673–2684. 10.1242/dev.02865 17592014

[B30] Jansen I. E. Savage J. E. Watanabe K. Bryois J. Williams D. M. Steinberg S.. (2019). Genome-wide meta-analysis identifies new loci and functional pathways influencing Alzheimer's disease risk. Nat. Genet. 51, 404–413. 10.1038/s41588-018-0311-9 30617256 PMC6836675

[B31] Krebs V. (2004). Books About Us Politics. Unpublished. Available online at: http://www.orgnet.com

[B32] Lancichinetti A. Fortunato S. Radicchi F. (2008). Benchmark graphs for testing community detection algorithms. Phys. Rev. E 78:046110. 10.1103/PhysRevE.78.046110 18999496

[B33] Langfelder P. Zhang B. Horvath S. (2008). Defining clusters from a hierarchical cluster tree: the Dynamic Tree Cut package for R. Bioinformatics 24, 719–720. 10.1093/bioinformatics/btm563 18024473

[B34] Lehri-Boufala S. Ouidja M.-O. Barbier-Chassefiére V. Hénault E. Raisman-Vozari R. Garrigue-Antar L.. (2015). New roles of glycosaminoglycans in α-synuclein aggregation in a cellular model of Parkinson disease. PLoS ONE 10:e116641. 10.1371/journal.pone.0116641 25617759 PMC4305359

[B35] Liberzon A. Birger C. Thorvaldsdóttir H. Ghandi M. Mesirov J. P. Tamayo P. (2015). The molecular signatures database hallmark gene set collection. Cell Syst. 1, 417–425. 10.1016/j.cels.2015.12.004 26771021 PMC4707969

[B36] Lin G. Wang L. Marcogliese P. C. Bellen H. J. (2019). Sphingolipids in the pathogenesis of Parkinson's disease and Parkinsonism. Trends Endocrinol. Metab. 30, 106–117. 10.1016/j.tem.2018.11.003 30528460

[B37] Lusseau D. Schneider K. Boisseau O. J. Haase P. Slooten E. Dawson S. M. (2003). The bottlenose dolphin community of doubtful sound features a large proportion of long-lasting associations. Behav. Ecol. Sociobiol. 54, 396–405. 10.1007/s00265-003-0651-y

[B38] Mäkinen V.-P. Civelek M. Meng Q. Zhang B. Zhu J. Levian C.. (2014). Integrative genomics reveals novel molecular pathways and gene networks for coronary artery disease. PLoS Genet. 10:e1004502. 10.1371/journal.pgen.1004502 25033284 PMC4102418

[B39] Martin B. De Maturana R. L. Brenneman R. Walent T. Mattson M. P. Maudsley S. (2005). Class II G protein-coupled receptors and their ligands in neuronal function and protection. Neuromol. Med. 7, 3–36. 10.1385/NMM:7:1-2:003 16052036 PMC2636744

[B40] Menche J. Sharma A. Kitsak M. Ghiassian S. D. Vidal M. Loscalzo J.. (2015). Uncovering disease-disease relationships through the incomplete interactome. Science 347:1257601. 10.1126/science.1257601 25700523 PMC4435741

[B41] Mihara T. Nakashima M. Kuroiwa A. Akitake Y. Ono K. Hosokawa M.. (2008). Natural killer cells of Parkinson's disease patients are set up for activation: a possible role for innate immunity in the pathogenesis of this disease. Parkinsonism Relat. Disord. 14, 46–51. 10.1016/j.parkreldis.2007.05.013 17702627

[B42] Mootha V. K. Lindgren C. M. Eriksson K.-F. Subramanian A. Sihag S. Lehar J.. (2003). PGC-1α-responsive genes involved in oxidative phosphorylation are coordinately downregulated in human diabetes. Nat. Genet. 34, 267–273. 10.1038/ng1180 12808457

[B43] Mosley R. L. Hutter-Saunders J. A. Stone D. K. Gendelman H. E. (2012). Inflammation and adaptive immunity in Parkinson's disease. Cold Spring Harb. Perspect. Med. 2:a009381. 10.1101/cshperspect.a009381 22315722 PMC3253034

[B44] Nalls M. A. Blauwendraat C. Vallerga C. L. Heilbron K. Bandres-Ciga S. Chang D.. (2019). Identification of novel risk loci, causal insights, and heritable risk for Parkinson's disease: a meta-analysis of genome-wide association studies. Lancet Neurol. 18, 1091–1102. 10.1016/S1474-4422(19)30320-5 31701892 PMC8422160

[B45] Nepusz T. Petróczi A. Négyessy L. Bazsó F. (2008). Fuzzy communities and the concept of bridgeness in complex networks. Phys. Rev. E 77:016107. 10.1103/PhysRevE.77.016107 18351915

[B46] Nicolas C. S. Amici M. Bortolotto Z. A. Doherty A. Csaba Z. Fafouri A.. (2013). The role of JAK-STAT signaling within the CNS. JAK-STAT 2:e22925. 10.4161/jkst.22925 24058789 PMC3670265

[B47] Parker W. D. Jr Parks J. K. Swerdlow R. H. (2008). Complex I deficiency in Parkinson's disease frontal cortex. Brain Res. 1189, 215–218. 10.1016/j.brainres.2007.10.061 18061150 PMC2295283

[B48] Peng J. Hui W. Li Q. Chen B. Hao J. Jiang Q.. (2019a). A learning-based framework for miRNA-disease association identification using neural networks. Bioinformatics 35, 4364–4371. 10.1093/bioinformatics/btz254 30977780

[B49] Peng J. Lu J. Hoh D. Dina A. S. Shang X. Kramer D. M.. (2020a). Identifying emerging phenomenon in long temporal phenotyping experiments. Bioinformatics 36, 568–577. 10.1093/bioinformatics/btz559 31304958

[B50] Peng J. Wang X. Shang X. (2019b). Combining gene ontology with deep neural networks to enhance the clustering of single cell RNA-seq data. BMC Bioinformatics 20:284. 10.1186/s12859-019-2769-6 31182005 PMC6557741

[B51] Peng J. Xue H. Wei Z. Tuncali I. Hao J. Shang X. (2020b). Integrating multi-network topology for gene function prediction using deep neural networks. Brief. Bioinformatics bbaa036. 10.1093/bib/bbaa036 32249297

[B52] Perozzi B. Al-Rfou R. Skiena S. (2014). “Deepwalk: Online learning of social representations,” in Proceedings of the 20th ACM SIGKDD international Conference on Knowledge Discovery and Data Mining (New York, NY: ACM), 701–710. 10.1145/2623330.2623732

[B53] Placido A. Pereira C. Duarte A. Candeias E. Correia S. Santos R.. (2014). The role of endoplasmic reticulum in amyloid precursor protein processing and trafficking: implications for Alzheimer's disease. Biochim. Biophys. Acta 1842, 1444–1453. 10.1016/j.bbadis.2014.05.003 24832819

[B54] Sharma A. Menche J. Huang C. C. Ort T. Zhou X. Kitsak M.. (2015). A disease module in the interactome explains disease heterogeneity, drug response and captures novel pathways and genes in asthma. Hum. Mol. Genet. 24, 3005–3020. 10.1093/hmg/ddv001 25586491 PMC4447811

[B55] Shi J. Malik J. (2000). Normalized cuts and image segmentation. IEEE Trans. Pattern Anal. Mach. Intell. 22, 888–905. 10.1109/34.868688

[B56] Su G. Kuchinsky A. Morris J. H. States D. J. Meng F. (2010). Glay: community structure analysis of biological networks. Bioinformatics 26, 3135–3137. 10.1093/bioinformatics/btq596 21123224 PMC2995124

[B57] Subramanian A. Tamayo P. Mootha V. K. Mukherjee S. Ebert B. L. Gillette M. A.. (2005). Gene set enrichment analysis: a knowledge-based approach for interpreting genome-wide expression profiles. Proc. Natl. Acad. Sci. U.S.A. 102, 15545–15550. 10.1073/pnas.0506580102 16199517 PMC1239896

[B58] Visscher P. M. Wray N. R. Zhang Q. Sklar P. McCarthy M. I. Brown M. A.. (2017). 10 years of GWAS discovery: biology, function, and translation. Am. J. Hum. Genet. 101, 5–22. 10.1016/j.ajhg.2017.06.005 28686856 PMC5501872

[B59] Wang M.-M. Miao D. Cao X.-P. Tan L. Tan L. (2018). Innate immune activation in Alzheimer's disease. Ann. Transl. Med. 6:177. 10.21037/atm.2018.04.20 29951499 PMC5994517

[B60] Wang S. Zhang S. Liou L.-C. Ren Q. Zhang Z. Caldwell G. A.. (2014). Phosphatidylethanolamine deficiency disrupts α-synuclein homeostasis in yeast and worm models of Parkinson disease. Proc. Natl. Acad. Sci. U.S.A. 111, E3976–E3985. 10.1073/pnas.1411694111 25201965 PMC4183298

[B61] Wang T. Peng J. Peng Q. Wang Y. Chen J. (2020). FSM: Fast and scalable network motif discovery for exploring higher-order network organizations. Methods 173, 83–93. 10.1016/j.ymeth.2019.07.008 31306744

[B62] Wang T. Peng Q. Liu B. Liu X. Liu Y. Peng J. Wang Y. (2019a). eQTLMAPT: fast and accurate eQTL mediation analysis with efficient permutation testing approaches. Front. Genet. 10:1309. 10.3389/fgene.2019.01309 31998368 PMC6970436

[B63] Wang T. Ruan J. Yin Q. Dong X. Wang Y. (2019b). “An automated quality control pipeline for eQTL analysis with RNA-seq data,” in 2019 IEEE International Conference on Bioinformatics and Biomedicine (BIBM) (San Diego, CA: IEEE), 1780–1786. 10.1109/BIBM47256.2019.8983006

[B64] Watanabe K. Taskesen E. Van Bochoven A. Posthuma D. (2017). Functional mapping and annotation of genetic associations with FUMA. Nat. Commun. 8:1826. 10.1038/s41467-017-01261-5 29184056 PMC5705698

[B65] Westra H.-J. Peters M. J. Esko T. Yaghootkar H. Schurmann C. Kettunen J.. (2013). Systematic identification of trans eQTLs as putative drivers of known disease associations. Nat. Genet. 45, 1238–1243. 10.1038/ng.2756 24013639 PMC3991562

[B66] Wu X. Reddy D. S. (2012). Integrins as receptor targets for neurological disorders. Pharmacol. Therap. 134, 68–81. 10.1016/j.pharmthera.2011.12.008 22233753 PMC3288359

[B67] Zachary W. W. (1977). An information flow model for conflict and fission in small groups. J. Anthropol. Res. 33, 452–473. 10.1086/jar.33.4.3629752

